# Spatial, environmental and anthropogenic effects on the taxon composition of hybridizing *Daphnia*

**DOI:** 10.1098/rstb.2008.0044

**Published:** 2008-06-02

**Authors:** Barbara Keller, Justyna Wolinska, Marina Manca, Piet Spaak

**Affiliations:** 1 Eawag, Swiss Federal Institute of Aquatic Science and Technology8600 Dübendorf, Switzerland; 2 Institute of Integrative BiologyETH Zurich, 8092 Zurich, Switzerland; 3 CNR Institute for Ecosystem Studies (ISE)28922 Verbania Pallanza, Italy

**Keywords:** hybrid superiority, environmental measures, coexistence, disturbance, eutrophication

## Abstract

The competitive ability of hybrids, compared with their parental taxa, can cover a wide fitness range from poor to superior. For example communities of the *Daphnia galeata*–*hyalina*–*cucullata* species complex often show hybrid dominance. We tested whether taxa composition of 43 European lakes inhabited by this species complex can be explained by habitat characteristics (e.g. size descriptors, trophy level) or geography. We found that *D. galeata* occurs more frequently south of the Alps, whereas *D. hyalina* and *D. cucullata* are found more in the north. Lakes with *D. galeata* dominance had higher temperatures whereas *D. hyalina* dominance could be attributed to low phosphorus loads. The dominance of F_1_-hybrids, however, was not explainable with current environmental variables. In a subset of 28 lakes, we studied the impact of eutrophication history on F_1_-hybrid success. Lakes with the highest trophic state in the past tended to be dominated by F_1_-hybrids. Our data demonstrate that human-mediated habitat disturbance (eutrophication) has facilitated hybrid success and altered the *Daphnia* taxon composition across lakes. At the same time, specific habitat conditions might provide a refuge from hybridization for native genotypes.

## 1. Introduction

Natural hybridization, resulting in crosses between genetically distinct populations of the same or different species (Arnold 1997), is a common phenomenon for species inhabiting both aquatic and terrestrial habitats (reviewed by Dowling & Secor 1997). Fitness of hybridizing taxa is influenced by environment-independent endogenous and environment-specific exogenous selection (reviewed by Burke & Arnold 2001). It has been shown that fitness of hybrids, compared with their parental species, can cover wide fitness ranges from poor to superior (reviewed by Arnold & Hodges 1995).

Three niche-based models exist to explain the coexistence of hybrid and parental species. The tension zone model (Barton & Hewitt 1985) assumes a balance between dispersal into the hybrid zone and selection against hybrids. By contrast, the bounded hybrid superiority model (Moore 1977) supposes that in intermediate environments, hybrids are fitter than either parental species. Other zones are known in which hybrids have a mosaic-like distribution within heterogeneous parental habitats (mosaic model, e.g. Harrison 1986). As such, each habitat patch has a unique set of environmental conditions, increasing the probability that natural selection can pick out the most fit genotypes, even though they might be rarely produced (Barton 2001). If the fitness of hybrids depends on environmental conditions, as the last two models propose, anthropogenic disturbance or fragmentation of natural systems will influence the structure of hybrid zones. A non-niche-based explanation of long-term coexistence is the neutral theory (Hubbell 2001), which assumes that species diversity is caused by drift. If species do show niche structures, the core assumption of the neutral theory is violated.

Hybrids that can propagate both sexually and asexually (e.g. plants with ramet production, cyclical parthenogenetic animals) can be especially successful owing to limited need for sexual reproduction, which otherwise causes serious problems in many hybrid taxa (i.e. recombination and segregation that break apart F_1_-hybrid genotypes; e.g. Emms & Arnold 1997). Cyclical parthenogens with regularly observed interspecific hybridization can be found within the genus *Daphnia* (Crustacea, Cladocera). In many natural *Daphnia* communities, hybrids coexist with their parental species (Wolf & Mort 1986; Taylor & Hebert 1992) and F_1_-hybrids are often the most abundant taxon (Taylor & Hebert 1992; Spaak & Hoekstra 1995; Hobæk *et al*. 2004). Spaak & Hoekstra (1995) proposed the temporal hybrid superiority model to explain long-term taxa coexistence and hybrid success in *Daphnia*. This model assumes that certain environmental conditions favour hybrids over their parental species and therefore ‘relative taxon fitness’ fluctuates in time with changing environments. *Daphnia galeata*, *Daphnia cucullata* and *Daphnia hyalina* (i.e. *D. galeata*–*hyalina*–*cucullata* species complex) and their hybrids are commonly found in sympatry in permanent lakes all across Europe (Spaak 1996; Schwenk & Spaak 1997). *Daphnia galeata* has a broad ecological niche, *D. hyalina* is most abundant in deep, oligo- to mesotrophic lakes, and *D. cucullata* has its core distribution in deep, moderately meso- to eutrophic lakes with average temperature and food availability (Flößner & Kraus 1986). Coexistence of parental species and hybrids can be explained by taxon-specific fitness differences indicated by different niche breadths (Weider 1993). *Daphnia* are sensitive to factors altered by the trophic state of lakes such as predation pressure (Spaak & Hoekstra 1995; Kerfoot & Weider 2004), food quantity (DeMott *et al*. 2001) and quality (Hairston *et al*. 1999). Biogeographic patterns in European lakes showed that parental species and hybrids occur in a rather patchy distribution with large differences in taxon compositions (Schwenk & Spaak 1997), and to date no clear habitat preferences for parental and hybrid classes have been formulated.

Many lakes experienced extensive trophy changes during the last century, which are known to disturb entire lake ecosystems (Schindler 2006). Disturbance has been described to facilitate the success and spread of hybrids (reviewed by Allendorf *et al*. 2001). The purpose of this study was to investigate the differences in *Daphnia* taxon composition and hybrid success across lakes with various habitat conditions, especially with different trophic histories. Specifically, we addressed the following questions: (i) how much of the variation in the taxon composition can be attributed to environmental and spatial variables (EV; SV); (ii) do *D. galeata*, *D. hyalina* and their F_1_-hybrids differ in responses to EV and SV; (iii) can the current *D. galeata*-, *D. hyalina*- and F_1_ dominance be explained by current EV and SV or by the past eutrophication.

## 2. Material and methods

### (a) Study sites and field sampling

Seventeen European lakes south and 26 lakes north of the Alps inhabited by the *D. galeata*–*hyalina*–*cucullata* species complex were screened for their *Daphnia* taxon composition (table 1). All the lakes are at elevations below 900 metres above sea level (m a.s.l.), except four lakes that are situated in an inner alpine Swiss valley above 1760 m a.s.l. Lakes were sampled in spring and autumn. Lakes south of the Alps were sampled twice (May 2004 and September 2004) and lakes north of the Alps were sampled two to four times (May–July 2003, August–October 2003 and August–October 2004). From three more intensively studied lakes (Brienzersee, Greifensee and Pfäffikersee) comparable sample dates were chosen. Samples of adult *Daphnia* with a body size of 1 mm or above (see Wolinska *et al*. 2007*a*) were collected using zooplankton nets with mesh sizes well below this threshold, so that all individuals of target size classes were collected (lakes south of the Alps: 126 μm mesh; lakes north of the Alps: 250 μm). The samples were taken at the area of maximum depth (DM; table 1) from the upper 50 m in deep lakes or, in shallower lakes, over the entire water column. Approximately 70–100 adult asexual females were genotyped per sample. The samples from spring and autumn were pooled because we were interested in the overall taxon composition. Taxon composition in hybridizing *Daphnia* populations may vary seasonally; however, annualized taxon composition remains rather stable over several years (Keller & Spaak 2004; De Meester *et al*. 2006).

### (b) Hybrid class identification

All the individuals were assayed at four polymorphic allozyme loci (AAT, AO, PGI, PGM) using cellulose acetate electrophoresis (see Keller & Spaak 2004). These loci were used to identify hybrid classes with NewHybrids (Anderson & Thompson 2002) that uses Bayesian statistics to calculate a posterior probability that reflects the level of certainty of an individual belonging to a specific hybrid category. Probability threshold for allocation was set to 95% or more; the remaining individuals could not be attributed with confidence to a specific hybrid class (F_*x*_) and therefore were excluded from subsequent statistical analyses. Since NewHybrids can only assign individuals to two hybridizing taxa, each lake population was divided into taxa pairs based on AAT and AO loci that are diagnostic for *D. galeata*, *D. hyalina* and *D. cucullata* (Wolf & Mort 1986; Gießler 1997). Individuals incorporating alleles from all three parental taxa (F_*x*_hgc) were excluded from the analysis with NewHybrids. In this way, we discriminated between *D. galeata* (Pgal), *D. hyalina* (Phyl), *D. cucullata* (Pcuc), *D. galeata*×*D. hyalina* F_1_-hybrids (F_1_hg), *D. hyalina*×*D. cucullata* F_1_-hybrids (F_1_hc), and *D. galeata*×*D. hyalina*×*D. cucullata* F_*x*_-hybrids (F_*x*_hgc).

### (c) Abiotic lake descriptors, trophic state and lake history

To analyse geographical distribution of the studied *Daphnia* communities, we used latitude (LA) and longitude (LO) as environmental descriptors. In addition the terms for a cubic surface regression (*xy*, *x*^2^, *y*^2^, *x*^2^*y*, *xy*^2^, *x*^3^ and *y*^3^) were applied because spatial species responses do not need to be linear (Borcard *et al*. 1992). Abiotic environmental descriptors were lake volume (VO), DM, surface area (SU), elevation (EL), temperature (TE) and current total phosphorus loads (PT; table 1). Total phosphorus values representing the trophic lake state are mean spring circulation measures, and were obtained from long-term water quality monitoring projects in Italy and Switzerland or from other published sources (table 1). For five lakes with no published PT, values were determined from integrative water samples of the upper 20 m. A comparison of field samples with literature data was done for 14 lakes; they did not differ significantly (Wilcoxon matched pairs test, *p*=0.42). As proxy for TE, we used the annual mean air temperature Climate Normals (1961–1990) from the Swiss Meteorological Institute (MeteoSwiss) corrected for sea level, which can be taken as surrogate for water temperature (Livingstone *et al*. 1999). Further, we were eager to know whether abiotic environmental and spatial parameters or trophic lake history might explain single taxon dominance. Therefore, we included in the analysis the highest total phosphorus load (PM) measured in the course of eutrophication (since 1900; obtained from the same sources as PT), the number of years since PM had occurred (YE) and the rate of phosphorus load change (Δ*P*) calculated as [log(PM/PT)]/Δ*t*. This analysis was applied to a subset of 28 lakes (hereafter referred as ‘historic dataset’; table 1).

### (d) Statistical analyses

Environmental parameters were log-transformed if needed. Since the *Daphnia* taxon dataset contains many zeros, we chose canonical correspondence analysis (CCA), a multivariate technique that considers only proportional relationships between variables and removes all differences between any data formats (see Jackson 1997). The CCAs as well as follow-up statistical tests (e.g. classical variation partitioning) were performed with the Canoco package, v. 4.5 (ter Braak & Šmilauer 2002). Variation partitioning with adjustments was performed using the program of Peres-Neto *et al*. (2006). Discriminant function analysis (DFA) and subsequent testing of selected variables with a Mann–Whitney *U*-test were performed with Statistica for Windows, release v. 7.1 (StatSoft, Inc.). Details of analyses are given below.

#### (i) Influence of EV and SV

CCA (Borcard *et al*. 1992) was used to partition the variance in the *Daphnia* taxa dataset into pure spatial, pure environmental and spatially structured environmental fractions. This was done in order to disentangle explanatory power attributed to each of these three fractions and to evaluate gradients and patterns found in the CCA. For this analysis, we discriminated between spatial (SV-variables: LA, LO, *xy*, *x*^2^, *y*^2^, *x*^2^*y*, *xy*^2^ and *x*^3^) and EV (VO, DM, SU, EL, TE and PT) variables. Data reduction was obtained with forward selection (ter Braak & Šmilauer 2002). A permutation test with 999 Monte Carlo permutations and *α*≤0.05 was used for the forward selection procedure and further to test the significance of variables in the full model CCA. Variance inflation factors (VIFs; a measure for cross-correlation of explanatory variables) were checked and eliminated if VIFs were more than 20 (threshold criterion for exclusion given by ter Braak & Šmilauer 2002). A partial CCA with selected EV- and SV-variables as covariables (covariable CCA) was performed to test the robustness of the pure EV fraction in explaining *Daphnia* taxon patterns. In addition, the explanatory significance of each of the two datasets (EV and SV) was tested with a new, unbiased variance partitioning method proposed by Peres-Neto *et al*. (2006).

#### (ii) Daphnia community description

A CCA including all selected EV- and SV-variables (full model CCA) was used to disentangle whether differences in *Daphnia* taxon composition can be attributed to environmental and spatial gradients. Statistical significance of the full model as well as the first two canonical axes was determined by a randomization test (999 permutations, Lepš & Šmilauer 2003).

#### (iii) F_1_hg-hybrid and parental success

To understand which factors determine taxon dominance, we discriminated between lakes where single taxon abundance was more than 50% (hereafter referred to as ‘dominated’) and the other lakes (‘not dominated’). Stepwise DFA (*α*=0.05, tolerance greater than 0.01) was performed to test which set of variables is able to discriminate between dominated and not dominated communities. An additional DFA was performed for F_1_hg-hybrids, to determine whether the variables of the trophic lake history (historic dataset; table 1) were powerful to discriminate between dominated and not dominated lakes. The ability of the forward selection procedure in DFA to find the best explanatory variable has been criticized (James & McCulloch 1990); therefore we tested the discriminatory power of the selected significant variables with a Mann–Whitney *U*-test.

## 3. Results

### (a) Influence of EV and SV

Variance partitioning was used to separate pure EV, pure SV and spatially structured EV variation in the *Daphnia* communities. With forward selection, PT, TE, SU and VO entered in the EV-CCA, whereas in the SV-CCA only LA was included (table 2). EV and SV-variables together (full model CCA) explained 34.8% of the variation in the *Daphnia* taxa dataset. This variance could be attributed with the method of Borcard *et al*. (1992) to 21.4% as pure EV, 4.3% as pure SV and 9.1% as spatially structured EV (table 2). In fact LA was the greatest single predictor of species occurrence. However, 65.2% of the variation remained unexplained. The covariable CCA showed that the pure EV-fraction was significant (*F*=3.04; *p*=0.002), with PT as the only significant variable (table 2). With the adjusted method of Peres-Neto *et al*. (2006), 9.9% of the variation could be attributed to pure EV, 2.4% to pure SV and 5.1% to spatially structured EV. Only the pure EV-fraction explained a significant portion of the variation in the dataset (*p*_EV_=0.001 and *p*_SV_=0.56).

### (b) *Daphnia* community description

Species occurrence and responses to gradients in the EV and SV were used for a description of *Daphnia* community patterns and to detect putative taxa-specific habitat preferences. In most lakes, hybrids co-occurred with one or two parental taxa (figure 1). Coexistence of hybrids with all three parental species was found in Lago di Iseo (no. 32). F_1_hg-hybrids were found in 25 lakes, F_1_hc-hybrids in 3 lakes, F_*x*_hgc-hybrids in 15 lakes and F_*x*_-hybrids in all lakes excluding Brienzersee (no. 4; figure 1). Twenty-six lakes were dominated by a single taxon (Pgal, 11 lakes; Phyl, 5 lakes; Pcuc, one lake and F_1_hg-hybrids, 9 lakes). Both canonical axes (CCA1: *F*=9.00, *p*=0.001 and CCA2: *F*=4.36, *p*=0.03) and the entire CCA (*F*=3.96, *p*=0.001) explained a significant amount of the weighted variance in the *Daphnia* taxon composition (figure 2). The randomization test indicated that PT, VO, TE and LA explain a significant amount of the variation in the species data (table 2). None of the final variables that entered the model had VIF larger than 20. Based on the full model CCA, we could discriminate between lakes north and south of the Alps. Phyl and Pcuc occurred more in larger, low phosphorus lakes and Pgal occurred more in warmer, more southern lakes. All hybrid classes clustered together, but not in an intermediate position to their parental species (figure 2).

### (c) F_1_hg-hybrid and parental success

To answer the question about which variables are responsible for single taxon dominance, we performed stepwise discriminant function analyses (DFAs). Only for Pgal and Phyl the DFA was significant. For Pgal dominance, TE was the only significant variable, whereas for Phyl dominance, PT was significant (table 3). With the fitted models several lakes were misclassified (Pgal: seven lakes; Phyl: four lakes; figure 3). F_1_hg dominance was not explainable with the recent dataset (DFA: Wilks' *λ*=0.806, *F*_4,38_=2.28, *p*=0.078) but with trophic lake history, in particular with PM (table 3). Only one lake was misclassified in this case (figure 3). The discriminatory power of all selected significant DFA variables was approved using Mann–Whitney *U* tests (Pgal_TE_, *p*<0.001; Phyl_PT_, *p*=0.021 and F_1_hg_PM_, *p*=0.019).

## 4. Discussion

Differences in the taxa composition of the studied *Daphnia* communities was more attributed to environmental variation than to spatial variation, using the classical method of Borcard *et al*. (1992) and the adjusted method of Peres-Neto *et al*. (2006). Taxon occurrence patterns were explained by EV (PT, SU, TE and VO). *Daphnia hyalina* dominance was attributed to present total phosphorus loads (PT) and *D. galeata* dominance was attributed to TE. F_1_hg dominance, however, was only explainable with historic phosphorus loads (PM; figure 3). This finding indicates that although lakes have recovered from past eutrophication, they are still affected by previous pollution.

Separation of *D. hyalina* and *D. galeata* along environmental gradients is consistent with biogeographic patterns (figure 1). For example, *D. hyalina* is more likely to be found in large lakes with low total phosphorus loads (figure 2) as described by Flößner & Kraus (1986). The total phosphorus load additionally explains the *D. hyalina* dominance, pronouncing the susceptibility of this taxon to eutrophication (table 3; figure 3). *Daphnia galeata* occurrence was associated with warm temperatures and low altitude (figure 2), whereof temperature was more important as it explains *D. galeata* dominance (table 3; figure 3). We did not find a significant effect of total phosphorus load on *D. galeata* dominance (table 3), although Flößner & Kraus (1986) stated that this species is promoted by eutrophication.

In contrast to *D. galeata*, F_1_hg-hybrids responded to the trophic conditions. Although present EV provided no satisfactory explanation of F_1_hg success, historic high trophic conditions significantly explained the present F_1_hg dominance (figure 3). Strong trophy changes can disturb entire lake ecosystems, by influencing the biomass, species richness and community composition of phytoplankton (e.g. Seip & Reynolds 1995). Disturbance leads to habitat changes or is the origin of novel habitat types (e.g. deep, cold eutrophic lakes), which hybrids then might occupy. Our study indeed suggests that *Daphnia* hybrids have novel ecological preferences: all hybrid classes clustered together in the multivariate space but not in the intermediate way to their parental species (figure 2). A similar pattern is reported from a recent experimental study: *Daphnia* hybrids' reaction norms were not intermediate to the ones of parental species (Wolinska *et al*. 2007*b*). Another factor that may facilitate hybrid persistence and success is asexual reproduction (e.g. Moody & Les 2002). Asexually reproducing hybrids can avoid or at least delay hybrid breakdown (the break up of co-adapted gene complexes), which allows fit genotypes to reach high abundances. High fitness of asexual *Daphnia* hybrid clones (Keller *et al*. 2007) may alter competition among taxa and hence may accentuate the influence of environmental factors on *Daphnia* populations.

Changes in ecosystems may alter the biology of species and thus enable hybridization by breaking down phenological barriers (Lamont *et al*. 2003). On the other hand, disturbance might lead to ill-adapted populations, increasing the chance for successful invasion and colonization (Allendorf *et al*. 2001; Levin 2004). In hybridizing species, the presence of hybrids in a specific habitat can be explained in two ways. Either hybrid *Daphnia* taxa are locally produced (Spaak 1997; Jankowski & Straile 2003; Keller & Spaak 2004; Keller *et al*. 2007), which requires coexistence of both parental species during certain time periods, or hybrids are produced elsewhere and subsequently colonize new biotopes. The first mechanism relies on dispersal of parentals, whereas the latter one relies on dispersal of hybrids. Dispersal of species from the *D. galeata*–*hyalina*–*cucullata* species complex is coupled with sexually produced diapausing eggs. Hybrids of this species complex, however, have a reduced sexual fitness (Keller & Spaak 2004; Keller *et al*. 2007), indicating a reduced dispersal capacity. By contrast, *D. galeata* invests more in sexual reproduction than hybrids (Keller *et al*. 2007) or *D. hyalina* (Jankowski & Straile 2004). This suggests that *D. galeata* may have invaded lakes that were formerly inhabited by other species and that hybrids were locally produced. This scenario is likely as *D. galeata* hybridizes with almost every species in the *D. longispina* complex (Taylor *et al*. 2005). Moreover, there is genetic evidence for multiple hybridization events in natural *Daphnia* populations (e.g. Spaak 1997).

Geographical barriers that have traditionally been considered as natural boundaries for dispersal, such as the Alps, have become porous due to human activity during the last century. Human transportation across Europe and the Alps was suspected, for example, to cause the spread and colonization of new biotopes by *D. parvula* (reviewed by Panov *et al*. 2004). When a species reaches a new water body, its capacity for colonization depends on a competitive advantage under given environmental conditions (De Meester *et al*. 2002). Abiotic and biotic conditions that promote coexistence of parentals and hybrids in natural *Daphnia* populations sometimes enable one taxon to dominate entire lake systems (figure 3). In addition to the conditions that we have tested in the present study, there are other important biotic factors able to maintain the coexistence of *Daphnia* taxa, for example, dynamic frequency-dependent host–parasite interactions (Wolinska *et al*. 2006) or delayed hybrid breakdown due to low sexual reproduction (Keller *et al*. 2007).

So far we have discussed species composition shifts based on trophic state changes, dispersal capacity of *Daphnia* and hybrid production. Other processes such as stochastic space–time fluctuations, and the noise introduced by unmeasured EV might interfere with EV and SV used to describe community structures in this study. Uncertainty factors may result in a large fraction of unexplained variations, which is indeed not uncommon in ecological surveys (e.g. Borcard *et al*. 1992; Lepš & Šmilauer 2003). But even in cases with explained variability less than 10%, well-interpretable structures can be detected (Lepš & Šmilauer 2003). In the deep oligotrophic Lago Maggiore (no. 34), for instance, a recent change in the *Daphnia* community composition from *D. hyalina* to *D. galeata* (Manca 2004) was proposed to have been caused by a water temperature increase (of approximately 1°C in the upper 50 m over the last three decades), and by the shift to an earlier and longer thermal stratification (Manca *et al*. 2007). This example demonstrates the sensitivity of lake ecosystems to climate change as suggested by Schindler (2006). However, the rough proxy we used for water temperature (weather station data) is accurate enough to discover such long-term processes. We found that lakes with *D. galeata* dominance experienced higher temperature than not dominated lakes, confirming that *D. galeata* might be favoured by climate change.

Based on the analysis of 43 lake populations, we conclude that the present taxon composition of the *D. galeata*–*hyalina*–*cucullata* species complex can be attributed to disturbance (eutrophication) and abiotic habitat conditions. Our results show that contemporary F_1_hg dominance is mainly the result of the magnitude of phosphorus load in the past.

## Figures and Tables

**Figure 1:**
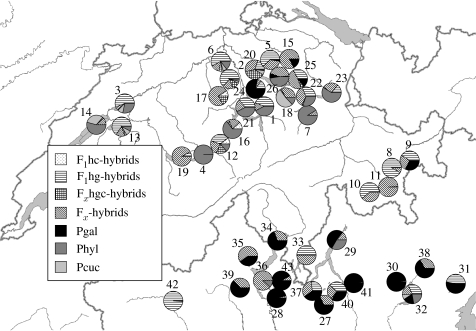
Taxon composition of *Daphnia* asexual females across 43 analysed lakes north and south of the Alps (2003 and 2004). Pie charts represent relative frequencies of three parental taxa (Pgal, *D. galeata*; Phyl, *D. hyalina*; and Pcuc, *D. cucullata*) and different hybrid classes (F_1_hg, *D. galeata*×*D. hyalina*; F_1_hc, *D. hyalina*×*D. cucullata*; F_*x*_hgc, *D. galeata*×*D. hyalina*×*D. cucullata*; and F_*x*_, later generation hybrids). For lake numbers see table 1.

**Figure 2:**
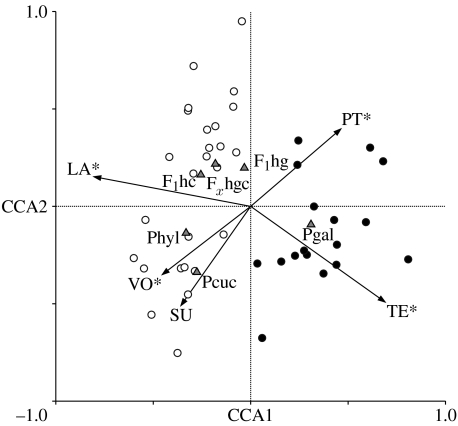
Full model CCA results of EV and SV on *Daphnia* communities in all 43 lakes north (open circles) and south (filled circles) of the Alps. Vectors represent environmental (VO, lake volume; PT, total phosphorus load; SU, lake surface; and TE, temperature) and spatial (LA, latitude) parameters that point in the direction of increasing importance for the respective variables. Arrow angles relative to axis and EV- or SV-variables indicate correlation strengths. Solid triangles symbolize relative proportions of various *Daphnia* taxa (Pgal, *D. galeata*; Phyl, *D. hyalina*; Pcuc, *D. cucullata*; F_1_hg, *D. galeata*×*D. hyalina*; F_1_hc, *D. hyalina*×*D. cucullata*; and F_*x*_hgc, *D. galeata*×*D. hyalina*×*D. cucullata*). Asterisks indicate variable explaining a significant amount of variation in the *Daphnia* taxa dataset (see table 2).

**Figure 3:**
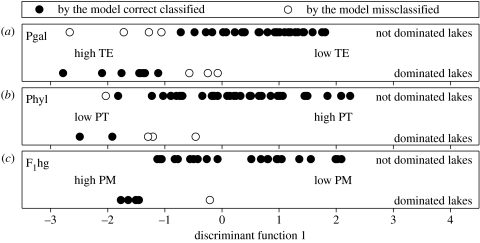
Distribution of ‘single taxon dominated’ (50% or more) and not dominated *Daphnia* communities along extracted first discriminant function: (*a*) *D. galeata* dominance (Pgal), (*b*) *D. hyalina* dominance (Phyl) and (*c*) F_1_hg dominance (F_1_hg). Discrimination for *D. galeata* and *D. hyalina* dominance was based on all 43 lakes and for F_1_hg dominance was based on 28 lakes with information about the trophic lake history (historic dataset; table 1). PT, total phosphorus load; TE, temperature; and PM, maximal phosphorus load.

**Table 1:** List with lake names, corresponding to the numbers in figure 1 and values of EV and SV. (Data sources: n.d., no data available; references (a) Federal Office for the Environment (FOEN; P. Liechti), (b) http://www.ise.cnr.it/limno/limno.htm, (c) BUWAL (2002), (d) Liechti (1994), (e) Osservatorio dei Laghi Lombardi (2005), (f) Ohlendorf (1998), (g) Elber *et al*. (2001), (h) Ludovisi *et al*. (2004), (i) Garibaldi *et al*. (1997), (j) Keller *et al*. (2002), (k) Keller (2003), (l) Ribi *et al*. (2001), (m) Kiefer (1987), (n) W. Steiner Nordostschweizerische Kraftwerke (NOK 2004, personal communication), and (o) field sampling June/July 2003, upper 20 m.)

corresponding no. (figure 1)	lake name	‘historic dataset’	total phosphorus load (PT, μg l^−1^)	maximal phosphorus load (PM, μg l^−1^)	years since PM (YE)	volume (VO; 10^6^ m^3^)	maximum depth (DM; m)	surface area (SU; km^2^)	elevation (EL; m a.s.l.)	longitude (LO)	latitude (LA)	references
*lakes north of the Alps*												
1	Ägerisee	X	10	23	29	353	83	7	724	8.618	47.127	a, d, k
2	Baldeggersee	X	52	517	28	173	66	5	463	8.258	47.207	a, c, d
3	Bielersee	X	13	140	33	1240	74	40	429	7.163	47.071	a, d
4	Brienzesee	X	3	25	22	5170	261	30	564	7.966	46.728	a, c, d
5	Greifensee	X	63	535	30	148	32	8	435	8.677	47.353	a, d, j
6	Hallwillersee	X	45	270	26	280	47	10	449	8.211	47.297	a, d
7	Klöntalersee		12	n.d.	n.d.	56	47	3	847	8.975	47.025	n, o
8	Lej da Champfèr		11	n.d.	n.d.	9	33	1	1791	9.806	46.469	f, o
9	Lej da S. Murezzan		43	n.d.	n.d.	20	44	1	1768	9.849	46.495	f
10	Lej da Segel		11	n.d.	n.d.	137	71	4	1797	9.732	46.418	f
11	Lej da Silvaplauna		13	n.d.	n.d.	127	77	3	1791	9.796	46.449	f
12	Lungenersee		10	n.d.	n.d.	66	68	2	689	8.162	46.804	l, o
13	Murtensee	X	27	155	23	550	46	23	429	7.105	46.940	a, d
14	Neuenburgersee	X	14	59	23	13 980	152	218	429	6.736	46.835	a, c, d
15	Pfäffikersee	X	22	379	32	59	35	3	537	8.783	47.352	a, d, g
16	Sarnersee	X	5	21	25	244	52	8	469	8.221	46.871	a, d
17	Sempachersee	X	34	165	19	639	87	14	504	8.158	47.143	a, c, d
18	Sihlsee		13	n.d.	n.d.	97	23	11	889	8.788	47.122	l, o
19	Thunersee	X	3	23	22	6470	217	48	558	7.673	46.713	a, d
20	Türlersee	X	15	157	28	6	22	0	643	8.503	47.270	g, l
21	Vierwaldstättersee	X	7	32	27	11 907	214	114	434	8.364	47.013	a, c, d
22	Wägitalersee		19	n.d.	n.d.	149	65	4	900	8.922	47.095	m, o
23	Walensee	X	2	30	27	3180	145	24	419	9.211	47.123	a, d
24	Zugersee	X	114	210	20	3174	198	38	413	8.483	47.161	a, d, l
25	Zürichsee, Obersee	X	14	41	31	467	48	20	406	8.845	47.203	a, d
26	Zürichsee, Untersee	X	25	130	33	3300	136	68	406	8.577	47.293	a, c, d
*lakes south of the Alps*												
27	Lago di Alserio	X	26	280	18	7	8	1	260	9.217	45.785	e, h
28	Lago di Comabbio	X	72	200	28	17	8	4	243	8.692	46.763	b, e, h
29	Lago di Como	X	35	74	24	22 500	410	145	198	9.267	46.000	e, h
30	Lago d'Endine	X	17	35	19	12	9	2	334	9.938	45.778	e, h, i
31	Lago d'Idro		24	n.d.	n.d.	684	122	11	370	10.517	45.767	b, e, h
32	Lago d'Iseo	X	17	41	23	7600	251	61	186	10.067	45.733	e, h
33	Lago di Lugano	X	47	176	23	5860	288	49	271	8.971	45.994	d, e, h
34	Lago Maggiore	X	11	35	25	37 500	370	213	194	8.654	45.967	a, c, e, h
35	Lago di Mergozzo		1	n.d.	n.d.	83	73	2	194	8.466	45.956	b, h
36	Lago di Monate		5	n.d.	n.d.	45	34	3	266	8.664	45.786	e, h
37	Lago di Montorfano	X	8	15	4	2	7	0	397	9.138	45.783	e, h
38	Lago Moro		8	n.d.	n.d.	4	42	0	389	10.158	45.863	b, e, h
39	Lago d' Orta		4	n.d.	n.d.	1300	143	18	290	8.400	45.817	b, h
40	Lago di Pusiano	X	74	200	12	69	24	5	259	9.273	45.802	e, h
41	Lago del Segrino		12	n.d.	n.d.	1	9	0	374	9.267	45.829	b, h
42	Lago di Sirio		24	n.d.	n.d.	5	44	0	271	8.929	45.451	b, h
43	Lago di Varese	X	82	400	28	160	26	15	238	8.750	45.800	e, h

**Table 2:** Explanatory contributions of EV and SV variables in canonical correspondence analyses (CCAs), determined by permutation test (999 Monte Carlo permutations and *α*≤0.05). (Full model CCA with all selected EV and SV variables; EV-CCA (pure EV- and spatially-structured EV-fraction); SV-CCA (pure SV- and spatially-structured EV-fraction), and covariable CCA (pure EV-fraction). Abbreviations: *λ*, explained variance; %, explained variance in percentages; LA, latitude; VO, lake volume; SU, lake surface; TE, temperature; PT, total phosphorus load. Marginal effects explain the variation in the species data singly, whereas conditional effects show the amount of extra variation each variable contributed when it was added to the models. For details see text.)

model type	variable	marginal (independent) effects				conditional (partial) effects			
	
		*λ*	*p*	*F*	%	*λ*	*p*	*F*	%
full model CCA	LA	0.30	0.001	6.53	13.3	0.30	0.001	6.35	13.3
	PT	0.14	0.022	2.80	6.2	0.15	0.008	3.28	6.6
	SU	0.11	0.075	2.16	4.9	0.09	0.059	2.24	4.0
	TE	0.27	0.002	5.52	12.0	0.12	0.026	2.69	5.3
	VO	0.13	0.051	2.44	5.8	0.13	0.011	3.13	5.8
EV-CCA	PT	0.14	0.023	2.80	6.2	0.12	0.024	2.85	5.3
	SU	0.11	0.073	2.16	4.9	0.18	0.002	4.14	8.0
	TE	0.27	0.001	5.52	12.0	0.27	0.001	5.52	12.0
	VO	0.13	0.038	2.44	5.8	0.12	0.017	2.68	5.3
SV-CCA	LA	0.30	0.001	6.35	13.3	0.30	0.001	6.35	13.3
covariable CCA	PT	0.15	0.012	3.24	6.6	0.15	0.012	3.28	6.6
	SU	0.09	0.111	1.84	4.0	0.10	0.053	2.24	4.4
	TE	0.10	0.085	2.11	4.4	0.11	0.035	2.69	4.9
	VO	0.08	0.143	1.74	3.5	0.12	0.009	3.13	5.3

**Table 3:** Variables that contribute to the discrimination of single taxon dominated *Daphnia* communities. (Variables were identified using a stepwise DFA with forward selection. The lower the Wilks' *λ* the higher is the discriminatory power of the entire model. The lower the partial *λ* the higher is the contribution of the variable to the overall discrimination. Discrimination for *D. galeata* and *D. hyalina* dominance was based on all 43 lakes and for F_1_hg dominance, it was based on 28 lakes with information about the trophic lake history (historic dataset; table 1). Pgal, *D. galeata*; Phyl, *D. hyalina*; F_1_hg, *D. galeata*×*D. hyalina*; LA, latitude; LO, longitude; VO, lake volume; SU, lake surface; TE, temperature; PT, total phosphorus load; PM, maximal phosphorus load.)

dominant *Daphnia* taxon	variable	Wilks' *λ*	partial *λ*	d.f.	*F*	*p*
Pgal	full model	0.623	—	3,39	7.88	<0.001
	TE	—	0.722	—	15.03	<0.001
	LO	—	0.866	—	6.03	0.019
	PT	—	0.975	—	1.00	0.322
Phyl	full model	0.769	—	3,39	3.91	0.016
	PT	—	0.831	—	7.93	0.008
	LA	—	0.943	—	2.37	0.132
	SU	—	0.966	—	1.37	0.248
F_1_hg	full model	0.652	—	2,25	6.68	<0.005
	PM	—	0.737	—	8.92	0.006
	LO	—	0.874	—	3.62	0.069
